# Supplementary material for: Causal interplay between lactose intolerance and gut microbiota: a combined bidirectional Mendelian randomization and in vivo validation study
Source: Front Nutr. 2026 Jun 1;13:1803337. doi: 10.3389/fnut.2026.1803337 (PMC13265576; doi:10.3389/fnut.2026.1803337)
Supplement: Supplementary file 1 [file Data_Sheet_1.zip › supplementary materials/Forward/leave one out cross validation/ebi-a-GCST90027762.finngen_R12_E4_LACTONAS.pdf]

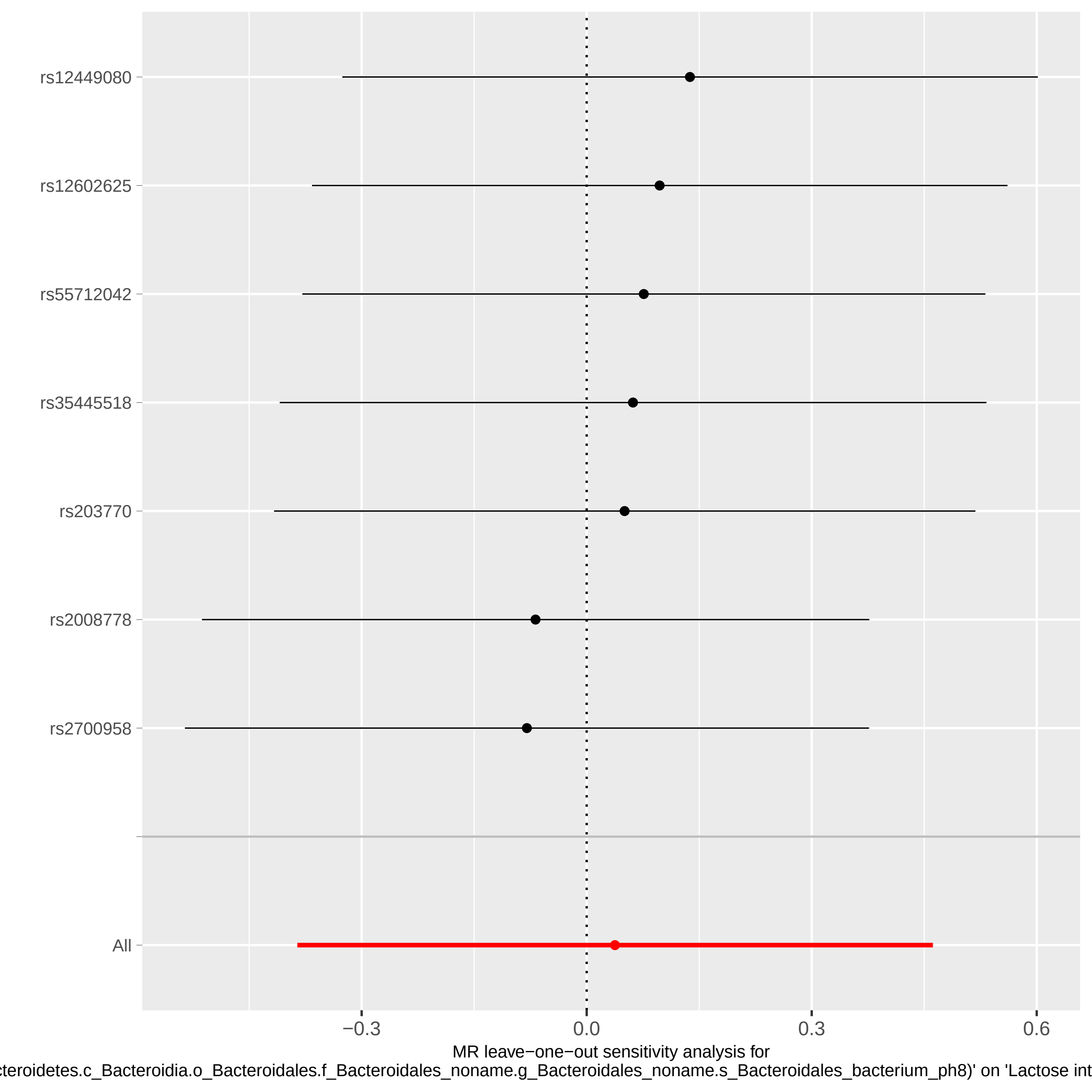
rs12449080
rs12602625
rs55712042
rs35445518
rs203770
rs2008778
rs2700958
All
−0.3
0.0
0.3
0.6
MR leave−one−out sensitivity analysis for
cteroidetes.c_Bacteroidia.o_Bacteroidales.f_Bacteroidales_noname.g_Bacteroidales_noname.s_Bacteroidales_bacterium_ph8)' on 'Lactose int
